# Effectiveness of accommodation and constant resistance training on maximal strength and power in trained athletes

**DOI:** 10.7717/peerj.441

**Published:** 2014-06-17

**Authors:** Jalil Ataee, Majid S. Koozehchian, Richard B. Kreider, Li Zuo

**Affiliations:** 1 Department of Physical Education and Exercise Science, Kharazmi University, Karaj, Iran; 2 The School of Health and Rehabilitation Sciences, The Ohio State University College of Medicine, Columbus, OH, USA; 3 Department of Kinesiology, Texas A&M University, College Station, TX, USA

**Keywords:** Muscle power, 1-Repetition maximum (1RM), Accommodation training, Constant training

## Abstract

Accommodation resistance is a training technique that may improve strength and power gains beyond those achieved by traditional free weights. In this method, chains are either added on a free-weight bar and combined with traditional plates or added to the bar as the entire load.

**Purpose.** The aim of the current study was to compare the effectiveness of accommodation and constant resistance training methods during a four-week period on maximal strength and power in trained athletes.

**Methods.** This study was comprised of 24 trained athletes, including 16 trained males [8 Wushu athletes (Kung-Fu) and 8 wrestlers, age: 20.5 ± 2.00 yrs. old]. Participants were initially tested on weight, body circumference, fat percent, upper and lower body maximal strength, determined by the 1-repetition maximum (1RM) test, which determines the greatest amount of weight a person can successfully lift, and upper and lower body power. Participants were equally randomized to either accommodation or constant resistance training groups. Both groups underwent resistance training for a four-week period that consisted of three sessions per week. Multivariate repeated-measures analyses of variance of the data were used to verify significant differences in strength and power between groups. The modified Bonferroni post hoc test was used to compare the obtained results in pre-, mid-, and post test.

**Results.** In the accommodation resistance group, there was a significant difference in lower body maximal strength compared to the constant group (163.12 ± 18.82 kg in the accommodation group vs. 142.25 ± 20.04 kg in the constant group, *P* = 0.04). No significant differences were found in upper body power, lower body power, and upper body maximal strength between the two groups (*P* > 0.05).

**Conclusion.** Although there was only a significant difference in lower body maximal strength between groups, accommodation resistance training may induce a physiological training response by improving the strength and power of stabilizing muscle groups required to balance the bar if consistently used over time.

## Introduction

Variable resistance training offers variable resistance throughout the entire range of motion (ROM) during exercise, whereas, constant resistance is a more traditional training protocol that demands unvarying resistance on the muscles and joints during an exercise routine. Chain accommodation training, a variable resistance technique, is becoming more popular among athletes as a possible alternative to traditional training methods. In this method, chains are either added on a free-weight bar and combined with traditional plates, or added to the bar as the entire load. The chain adding method at the end of a barbell was first popularized in sports clubs, bodybuilding centers, and strength colleges (Berning, Coker & Adams, 2004; Coker, Berning & Briggs, 2006; Souza, Shimada & Koontz, 2002). Berning et al. discussed how anecdotal support for the use of chains for strength and power enhancement has been mounting (Berning, Coker & Adams, 2004). Despite the prevalence of theories about accommodation resistance training, only a few experimental studies have been conducted on this type of training (Berning, Coker & Adams, 2004; Ghigiarelli et al., 2009). In addition, the efficacy of this training method over other training protocols has been controversial. Coker et al. found no significant difference when comparing chain loaded and traditional resistance in the Olympic clean exercise (Coker, Berning & Briggs, 2006). Nevertheless, chain accommodation resistance training should be further explored as a means to enhance an athlete’s strength and power.

Previous research suggests that chain accommodation training has been more effective than conventional free weight training at increasing strength and power (Berning, Coker & Adams, 2004; Ebben & Jensen, 2002; Simmons, 1996). Numerous studies provide evidence of the mechanical advantage supplemented by variable resistance training (Berning, Coker & Adams, 2004; Coker, Berning & Briggs, 2006; Kauhanen, Hakkinen & Komi, 1989). For example, during bench press when the bar is nearest to the chest (eccentric phase), muscles are at their longest and least stable state. Furthermore, the force exerted by the muscles varies throughout a joint’s ROM creating a mechanical advantage at various joint angles. There is an ascending strength curve throughout the ROM for the bench press that can be taken advantage of by using a variable resistance training protocol (Berning, Coker & Adams, 2004).

Since many sports require a combination of both muscular power and endurance, evaluation of the maximal strength level is important to assess the muscular power component. More importantly, power cannot reach higher standards without an increase in maximal strength (Bompa & Haff, 2009). In fact, the rate of recruitment and the activation velocity in motor units increases by applying higher loads and faster contractions (Berning, Coker & Adams, 2004). In addition to physiological factors, the maximal strength that an athlete can produce also depends on biomechanical characteristics of motion such as leverage and the joint angle (McCurdy et al., 2008).

Although accommodation resistance training may provide promising results, it appears that variable resistance devices cause a higher fatigue response compared to the constant resistance devices (Coker, Berning & Briggs, 2006; Keohane, 1986). This challenge is due to higher neural involvement during variable resistance activities compared to the constant resistance activities. Specifically in chain accommodation training, additional stabilization muscles are needed to steady the bar when the chains are completely lifted off the floor (McCurdy et al., 2008). It is possible that variable resistance activities are more suitable for highly trained athletes who need higher stimulation levels, as indicated by popularization of accommodation resistance training methods in sports clubs, bodybuilding centers, and strength colleges (Berning, Coker & Adams, 2004; Coker, Berning & Briggs, 2006; Ghigiarelli et al., 2009; Kauhanen, Hakkinen & Komi, 1989; Souza, Shimada & Koontz, 2002). Because of the high competition frequency and short-term preparation during the off-season in many sports, developing an appropriate training protocol to improve strength and power in a relatively short time period is necessary (Eckerson et al., 1998).

Many similarities exist between wrestling and Wushu (Kung Fu) with respect to energy systems, competing time period (three 2-min rounds), muscles involved in the activity, and the necessity for exceptional strength and power. Chain accommodation training may be an effective way to rapidly increase strength and power considering the mechanical advantage throughout the concentric phase of the exercise. In addition, the need for neural involvement and additional stabilizing muscles during chain accommodation training may enhance a trained athlete’s maximal strength and power. Therefore, this study evaluated the efficacy of chain accommodation resistance training on enhancing trained athlete’s muscle strength and power.

## Methods

This study was approved by the University of Kharazmi Human Subjects Committee (approval #: 3RT-0073). Forty trained males volunteered to take part in the study. Sixteen males [8 Wushu (Kung-Fu) athletes and 8 wrestlers] at age: 20.5 ± 2.00 yrs; height: 174.34 ± 6.53 cm; weight: 70.22 ± 10.50 kg; and fat percentage: 12.87% ± 4.23 were randomly selected to participate in the study. Participants were initially tested on maximal strength and power variables. One repetition maximum (1RM) test was used to measure isotonic muscle strength. One-RM bench press and squat testing were conducted to determine upper and lower body strength using procedures previously described (Kraemer & Fry, 1995). Participants performed a warm-up including 8–10 repetitions using a light weight, 3–5 repetitions using a moderate weight, and 1–3 repetitions using a heavy weight. After the warm-up sets, participants were tested for lRM strength by increasing the resistance on subsequent attempts until the participant was unable to complete an attempt using appropriate technique through a full ROM. Each attempt was separated by 3–5 min of rest. The upper body power was determined by throwing a 3.63 kg medicine ball. The medicine ball throw test is a valid test for assessing power for an analogous total-body movement pattern and general athletic ability (Stockbrugger & Haennel, 2001). The lower body power was assessed using a vertical jump test. All participants performed a 5-min warm-up (pedaling at 60 rpm at 300 kg m min^-1^ interspersed with five all-out sprints during the last 5-s of each minute) on a cycle ergometer prior to testing. Following the warm-up the participants performed two countermovement vertical jumps. The higher of the two trials was recorded (Hoffman et al., 2007). The counter-movement vertical jump height was measured by a Vertec (Sports Imports, Columbus, OH). Prior to testing, each participant’s standing vertical reach height was identified. Vertical jump height was calculated by subtracting the standing reach height from the jump height. Power outputs were calculated according to the formula of Harman and colleagues (Harman et al., 1991). Body circumference (chest, arm, waist, hip, and calf) was measured by a tape measure, and a three-point fat percent test (chest, arm, and thigh) was assessed using a caliper (Eckerson et al., 1998; Gore, 2000). Afterwards, the participants were randomly assigned into either the accommodation or the constant resistance training group and trained for a four-week period, with three sessions per week.

Utilization of loads exceeding 80% of 1RM during a special preparation phase is necessary to increase maximal strength (Bompa & Haff, 2009). Therefore, training in both groups including squats and bench presses were performed at 85% of 1RM in three sets, with five repetitions per set. Increasing the load using chains was continued until all of the chains were lifted up nearly 5 cm above the ground. The training period was 4-weeks, with three sessions per week in both groups. The training session for both groups was composed of warm-up, specific training, and cool-down. The total warm-up duration was 15 min, which included jogging (5 min) and dynamic stretch movements (10 min) as well as performing the bench press and squats with a barbell bar (20 kg) in 10 repetitions. During specific training, all participants performed the training at 85% of their 1RM in 3-sets with 5-reps and two minutes rest in between each set (Bompa & Haff, 2009). However, there was only one difference that occurred in the accommodation group, in which 20% of 1RM was gradually added to the load by chains along the range of motion (each 10° is 2.22% from 20% of 1RM along the 90° range of motion). A reference marker was provided for the participants during the squat movements to ensure proper depth of squats. The speed of movement was controlled for each exercise using a metronome (4 s per one complete squat movement). Participants in this study were prohibited from performing any type of resistance training outside of the required training sessions in order to control the efficacy of the training protocols. At the end of the training period, a retest was applied on the dependent variables to compare the performance between groups. In order to take part in competitions, wrestlers and Wushu (Kung Fu) athletes are required to maintain and control their weight; therefore, controlling participants’ diets was not possible, which was the major limitation of the study.

**Figure 1:**
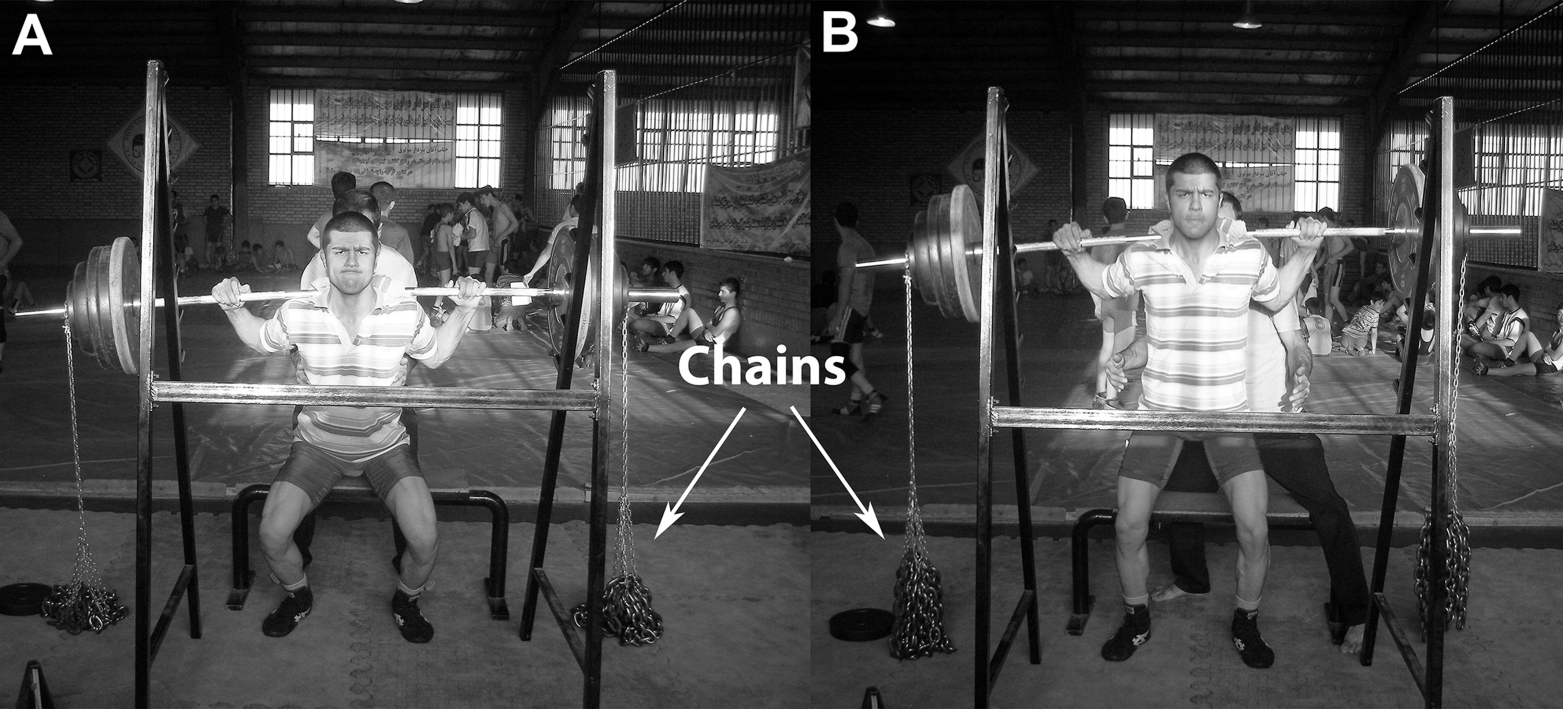
Training in accommodation resistance style. (A) Start; (B) chain link lifted.

The training load during accommodation training was variable between 85% of 1RM at the lowest and weakest muscle point and 100% of 1RM at the highest and strongest muscle point. When the barbell bar and its weights were lifted up, the links were also lifted up from the ground simultaneously and when the barbell and its weights were lowered, chain links dropped to the ground. Thus, the training load was increased or decreased in this way (Fig. 1). In this method, the whole 20% of chain load was lifted up about 5 cm from the ground when the joint was fully extended. The load was constant throughout the ROM (85% of 1RM) in the constant training group. Additionally, the speed of the movement was controlled by a metronome in both groups (each movement in 4 s).

### Statistical analyses

Descriptive and inferential statistics were used to analyze the data. The mean ± SD indices were used in descriptive statistics. Afterwards, the data were checked for normality using the Kolmogorov–Smirnov test. Analysis of covariance was used to compare the variables’ improvements in both groups. The significant difference level was set at *P* < 0.05. All statistical analyses were performed using SPSS for Windows (Release 22.0; Chicago, IL, USA).

## Results

The results of the analysis of covariance indicated that there was a significant difference in lower body maximal strength compared to the constant group (163.12 ± 18.82 kg in the accommodation group vs. 142.25 ± 20.04 kg in the constant group, *F* = 11.85, *P* = 0.04). However, there was no significant difference between the two groups in weight (*F* = 0.78, *P* = 0.39), body circumference (F = 2.42, P = 0.14), fat percent (*F* = 0.71, *P* = 0.41), upper body power (F = 1.34, P = 0.26), lower body power (*F* = 1.43, P = 0.25), and upper body maximal strength (*F* = 0.06, *P* = 0.80) (Table 1). Furthermore, the evaluation of effect size (ES) between groups indicated that the ES in weight (ES = 0.47), body circumference (ES = 0.83), fat percent (ES = 0.45), upper body power (ES = 0.45), lower body power (ES = 0.64), upper body maximal strength (ES = 0.13), and lower body maximal strength (ES = 1.84) were improved in the accommodation vs. the constant resistance training group.

**Table 1:** The statistical results of variables.

Variables	Groups	Measurement time		*F*	*P*	Effect size
		Pre test	Post test			
Weight (kg)	Accommodation	72.72 ± 13.32	72.82 ± 11.39	0.78	0.39	0.47
	Constant	67.67 ± 6.62	67.97 ± 5.69			
Body circumference (cm)	Accommodation	313.00 ± 29.27	319.81 ± 22.48	2.42	0.14	0.83
	Constant	313.87 ± 15.18	300.50 ± 34.72			
Fat %	Accommodation	13.53 ± 4.41	12.11 ± 4.93	0.71	0.41	0.45
	Constant	12.21 ± 4.22	11.85 ± 3.70			
Upper body power (m)	Accommodation	4.73 ± 0.41	5.06 ± 0.45	1.34	0.26	0.62
	Constant	4.56 ± 0.43	4.56 ± 0.47			
Lower body power (Watt)	Accommodation	4,055.87 ± 791.19	4,324.30 ± 746.32	1.43	0.25	0.64
	Constant	3,644.29 ± 394.02	3,814.66 ± 486.00			
Upper body strength (kg)	Accommodation	80.00 ± 10.84	92.50 ± 14.41	0.06	0.80	0.13
	Constant	76.50 ± 15.14	87.87 ± 16.68			
Lower body strength (kg)	Accommodation	117.62 ± 11.99	163.12 ± 18.82	11.85	0.04	1.84
	Constant	116.75 ± 24.02	142.25 ± 20.04			

## Discussion

The results of a 4-week training protocol, and its effectiveness in the accommodation and constant resistance training groups (Wushu athletes and wrestlers) on weight, body circumference, fat percent, upper and lower body power, and maximal upper body strength did not show any significant differences between groups (*P* > 0.05). However, a significant difference was seen in the maximal lower body strength between groups (*P* = 0.04), possibly due to more muscle mass in the legs compared to that in the chest. The value of the effect size has to be considered when evaluating this data because the actual meaningfulness (increase in strength/power) between groups is demonstrated.

Although the variables’ differences between groups were not statistically significant in the current study, the comparison of ES between groups indicates that maximal strength and power had a greater magnitude in the accommodation resistance group. This indicates that both upper and lower body maximal strength and power gains were significantly higher in the chain accommodation group. This can be attributed to the inability of traditional methods to increase force output near the end of the ROM due to additional resistance (McCurdy et al., 2009). Another salient point of this study was the necessity for stabilizing muscles, and further neural involvement near the end of the concentric phase of the exercise. This also may have had an impact on the higher ES in the accommodation group.

Maximal lower body strength was the only variable to elicit a significant difference from the analysis of covariance. Rhea et al. suggested that another variable to increase the rate of force development from resistance training (combination of free weights and elastic bands training) is the exploitation of the stretch–shortening cycle of muscle tissue (Rhea, Kenn & Dermody, 2009). The working skeletal muscle stores elastic potential energy during the eccentric phase of the lift and then releases this energy as a kinetic energy during the concentric phase of the lift. Wallace et al. suggested that rate of force development could be increased as a result of a longer peak velocity phase observed with variable resistance training (Wallace, Winchester & McGuigan, 2006). This is a characteristic that can be seen in the accommodation resistance training. Due to an increase in resistance through enhancing mechanical advantage, an athlete is able to generate the highest force levels during concentric phase of the lift, when muscles are at or near their optimal length–tension relationship.

The findings of our study are consistent with those of Dobbs et al., which observed a significant increase in ES of strength and vertical jump during 5-weeks of accommodation training, compared to the constant training (Dobbs, 2010). It is concluded that accommodation training is an appropriate method to increase lower body strength. However, the observed changes in strength and power are most likely due to neuronal adaptations and innervation from Type I to Type II fibers. The change in innervation allows for recruitment of faster and more powerful muscle fibers (Gabriel, Kamen & Frost, 2006). Alternatively, in a separate study by Ebben and Jensen, the loading pattern was conducted by including the total load using chains and bands (Ebben & Jensen, 2002); therefore, the constant load was decreased.

In addition, the accommodation resistance training improves maximal leg strength more than regular strength training. Additional studies should be conducted in order to obtain well rounded insight into the importance of accommodation resistance training. Further research should include athletes with varying training backgrounds to observe muscle and nervous system adaptation, as well as improvement in their specific sport. Research into long term effects of accommodation resistance training on fitness of joints as well as overall fitness will be extremely beneficial and provide long term outcome of such a vigorous training method. That being said, this training form is as effective as regular strength training and may be used for those individuals that prefer accommodation training and may therefore even get better improvements.

## Supplemental Information

10.7717/peerj.441/supp-1 Supplemental Information 1 Raw Data
